# Metagenomics for antimicrobial resistance: from resistome surveillance to mechanistic inference

**DOI:** 10.1128/jb.00090-26

**Published:** 2026-07-14

**Authors:** Jingyu Cao, Zheng Ye, Jiangang Pan

**Affiliations:** 1 School of Life Science and Technology, Inner Mongolia University of Science & Technology, Baotou, China; 2 Institute of Computational Science and Technology, Guangzhou University 47875, Guangzhou, China; University of Massachusetts Chan Medical School, Worceste, Massachusetts, USA

**Keywords:** antimicrobial resistance, metagenomics, antibiotic resistance genes, mobile genetic elements, horizontal gene transfer, clinical metagenomics

## Abstract

Antimicrobial resistance (AMR) is a global health crisis shaped by complex ecological and evolutionary processes that often occur in polymicrobial communities. Metagenomics enables culture-independent profiling of microbial DNA directly from clinical or environmental samples, providing an unparalleled view of community composition, resistome content, and the mobile genetic elements that drive horizontal gene transfer (HGT). Yet, a recurring challenge is that metagenomic detection of antibiotic-resistance genes does not automatically translate into a mechanistic understanding of resistance phenotypes, nor does it replace culture-based functional validation. Here, we synthesize how modern metagenomics supports AMR research across three linked questions: (i) what resistance determinants are present and how do they change across time and space, (ii) which hosts and mobile genetic elements carry these determinants, and how gene flow can be inferred, and (iii) what evidence is required to move from “resistance potential” to robust mechanistic claims. We emphasize practical design principles (sampling, controls, and contamination management), analytical choices (database and parameter effects), and recent advances, including long-read sequencing for resolving antibiotic-resistance genes context, and rapid clinical metagenomic sequencing for time-sensitive decision support. We propose an evidence ladder for mechanistic inference that integrates metagenomics with targeted assays and culture-dependent experiments. Beyond synthesizing recent advances, this review provides operational tools for critical appraisal and study design: an evidence ladder for mechanistic inference, a decision-gated workflow that ties metagenomic outputs to allowable claim language, a minimum reporting checklist aligned to evidence strength, and a “pitfall → consequence → fix” guide to reduce over-interpretation. To support a more comprehensive, forward-looking view, we also summarize emerging directions that are rapidly reshaping AMR metagenomics—multi-omics integration, single-cell, and epigenetic linkage strategies, CRISPR-enabled enrichment/depletion, and AI-assisted discovery/mining—and clarify where these advances strengthen (or do not strengthen) mechanistic claims within the same evidence ladder.

## INTRODUCTION

### Why antimicrobial resistance needs community-level genomics

Antimicrobial resistance (AMR) threatens modern medicine and imposes a growing burden on health systems worldwide (1, 2). Classic microbiology and antimicrobial discovery were built on cultivation, isolation, and phenotyping (3). These approaches remain indispensable: they define susceptibility, reveal physiology, and enable causal testing. However, culture-dependent methods alone can undersample the uncultured fraction of microbial communities and can miss community interactions that shape resistance emergence and persistence (4).

Metagenomics—sequencing DNA directly from complex samples—complements cultivation by enabling community-scale surveillance and by capturing genetic context that is difficult to obtain from isolates alone (5–7). In AMR studies, metagenomics is most powerful when it answers clearly framed questions and when claims about “mechanisms” are linked to evidence beyond gene detection.

### Scope and contributions of this review

**Fig 1:**
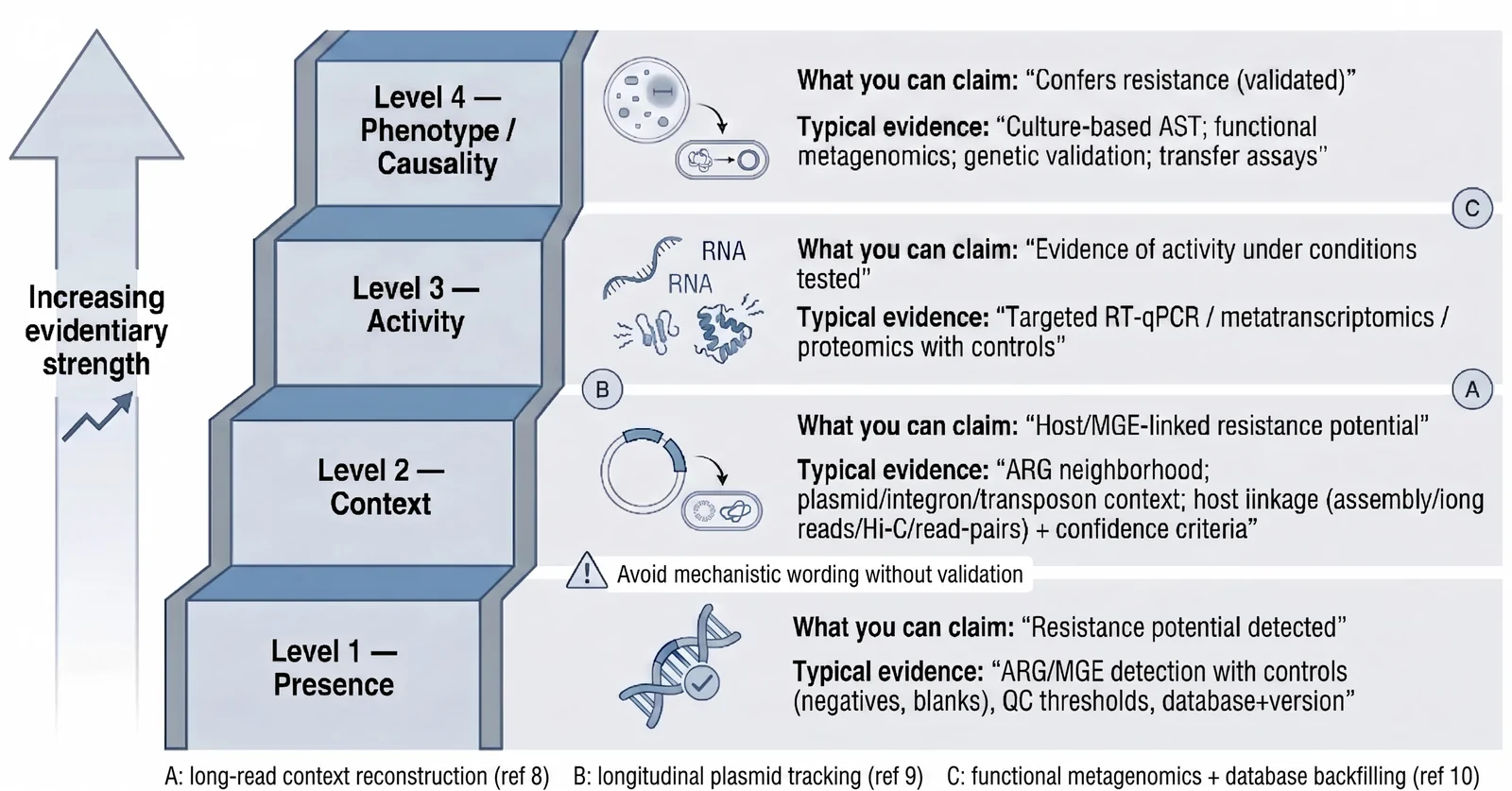
Evidence ladder for mechanistic inference in metagenomic AMR studies. Schematic of four evidence levels, from Level 1 (antibiotic-resistance genes/mobile genetic elements [MGEs] presence with controls) to Level 4 (phenotype/causality demonstrated by culture-dependent or functional assays). The figure highlights typical data sources and the strongest appropriate wording at each level (e.g., “resistance potential” at Levels 1 and 2 versus “confers resistance” at Level 4). In-figure callouts map the ladder to representative studies: A: Level 2 context from long reads [8], B: Levels 2 and 3 context with longitudinal/plasmid tracking [9], and C: Level 4 phenotype-anchored discovery and database backfilling [10].

**Fig 2:**
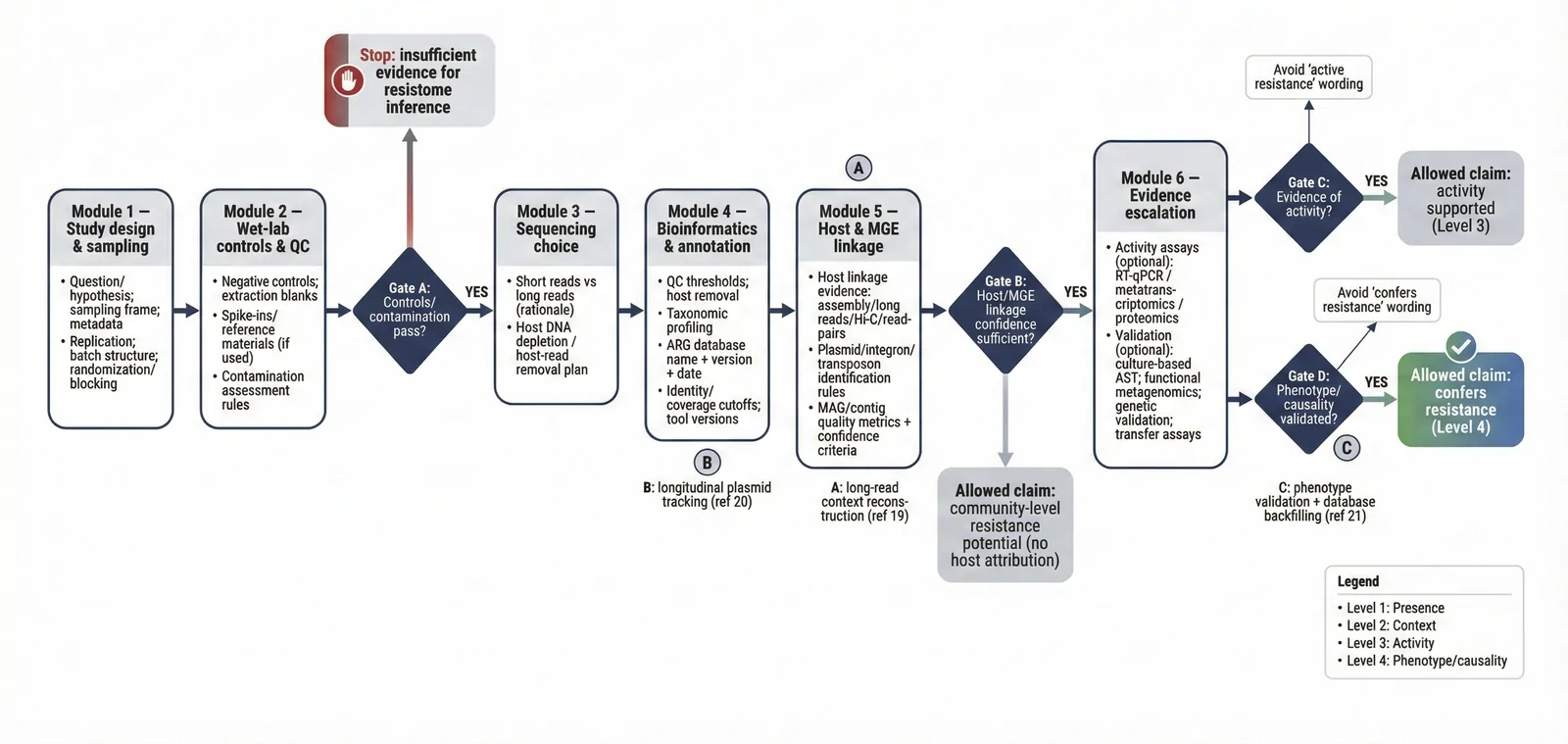
A workflow with decision gates linking metagenomic outputs to allowable AMR claims. End-to-end overview from sampling and laboratory controls (negative controls, extraction blanks, and spike-ins/reference materials) through sequencing (short reads versus long reads), analysis (QC, host removal, taxonomic profiling, and antibiotic-resistance genes annotation with database versioning), and host/mobile genetic elements linking. In-figure callouts align workflow stages to the case studies in “Representative Case Studies: What Metagenomics Uniquely Enables”: **A** highlights long-read context reconstruction near Module 5/Gate B (8), **B **highlights time-series/plasmid tracking spanning Module 1 and Module 5 (9), and **C** highlights phenotype validation and database backfilling near Module 6/Gate D (10).

This review focuses on shotgun metagenomics for AMR with a specific goal: improving the rigor of mechanistic interpretation (i.e., moving from detection to defensible claims about context, activity, and phenotype). While the central contribution remains an interpretation framework (the evidence ladder; Fig. 1) paired with a decision-gated workflow (Fig. 2) and operational checklists (Tables 1 and 2), we also broaden the perspective to cover key enabling technologies and analytic frontiers (see Enabling Technologies and Analytical Frontiers (Toward a More Comprehensive Review)) that increasingly shape what is feasible in practice. To keep the framework grounded, Fig. 1 and 2 include in-figure callouts (A–C) that tie evidence escalation to representative high-impact studies.

**TABLE 1:** Minimum reporting checklist and reviewer-checkable bundle for metagenomic AMR studies aiming for mechanistic inference

Module/evidence goal	Minimum to report in text (methods/results)	Reviewer-checkable deliverables (main text/supplement/repository)	Robustness checks (recommended)
Study design (all levels)	Question/hypothesis; sampling frame; metadata (time/location/exposure); replication; batch structure; randomization/blocking; intended claim level (Levels 1–4)	Sampling diagram; cohort/metadata table; analysis plan summary	Re-run key comparisons with/without batch covariates; report missingness/imbalance
Wet-lab controls and contamination handling (Level 1)	Negative controls (extraction/library blanks); positive controls/reference materials (if used); contamination assessment and filtering rules	Control read summaries; contaminant list/rules; detection limits for low biomass	Show conclusions under alternative contamination thresholds
Sequencing strategy and host background (Levels 1 and 2)	Platform choice (short/long/hybrid); depth targets; host depletion/removal plan; software + thresholds for host read removal	Read budget + host fraction summary; yield/QC metrics	Downsampling analysis (Do conclusions hold at lower depth?)
Antibiotic-resistance genes calling (Level 1)	Antibiotic-resistance genes database name + version + date; tool versions; identity/coverage cutoffs; multi-mapping rules	Antibiotic-resistance genes tables; resistome profiles; parameter/config snapshot	Compare at least one alternative database/threshold set; report concordance
Quantification across samples (Levels 1 and 2)	Normalization strategy (per biomass/per cell equivalents/per volume/per 16S); compositional handling; uncertainty measures	Differential abundance outputs with uncertainty; normalization rationale	Sensitivity to alternative normalization schemes; report stable vs unstable findings
Taxonomic profiling/genome-resolved reconstruction (Levels 1 and 2)	Classifier/aligner + database version; assembly/binning approach; MAG^*a*^ quality metrics (completeness/contamination); decision rules	Taxa profiles; MAG statistics; binning QC summary	Alternative profiler/classifier or alternative assembly/binning parameters
Host–antibiotic-resistance genes/mobile genetic elements linkage (Level 2)	Linkage signal type(s) (spanning long reads/read pairs/Hi-C/methylation); explicit thresholds; plasmid/integron/transposon identification rules; linkage confidence criteria	Linkage evidence table: spanning-read counts + span length; read-pair counts + insert/orientation checks; Hi-C statistic + threshold (if used); linkage retention under alternative assemblies/filters	Quantify how often top linkages change under reasonable re-assemblies/filters
Gene flow/dissemination inference (Levels 2 and 3)	Define “potential mobility” vs “observed transmission”; criteria for calling transfer vs clonal spread; time-series/strain tracking rules; epidemiological linkage (if clinical)	HGT^*a*^ candidate list with criteria; longitudinal plots	Conservative language when uncertain; strain-resolved validation when feasible
Activity evidence (Level 3; optional)	Assay type; conditions; controls; mapping rules; condition specificity	Expression/activity readouts linked to conditions	Condition-matched replication; avoid generalizing beyond tested conditions
Phenotype anchoring (Level 4; optional)	Validation route (culture AST/functional metagenomics/genetics/transfer assays); mapping of genotype → phenotype; reproducibility	AST results linked to the determinant; functional/transfer assay outputs	Independent validation route or replicate experiments

^*a*^ HGT, horizontal gene transfer; MAG, metagenome-assembled genome.

**TABLE 2:** Common pitfalls that inflate mechanistic claims in metagenomic AMR studies (and minimum fixes)

Pitfall	Why it matters	Minimum fix (what to report/do)	Evidence ladder impact
Gene presence described as “resistance mechanism” or phenotype	DNA-based detection does not guarantee expression, functionality, or clinical resistance	Use calibrated language (“resistance potential”); add validation plan and cite supporting phenotype evidence if available	Inflates Level 1 → Level 4
Resistome differences reported without clear normalization/compositional handling	Relative abundance shifts can be artifacts of biomass changes or compositional constraints	State normalization (per biomass/per cell equivalents/per volume); report uncertainty and sensitivity analyses	Weakens Level 1 comparisons
Host/antibiotic-resistance genes linkage claimed without specifying evidence type and confidence criteria	Misassigned hosts drive incorrect conclusions about dissemination and reservoirs	Specify linkage method (long reads/assembly/read-pairs/Hi-C) and thresholds; report MAG/contig quality metrics	Inflates Level 1 → Level 2
“HGT occurred” claimed from mobile genetic elements signatures alone	Mobility potential is not transmission; epidemiology/time-series is needed for directionality	Distinguish “potential mobility” vs “observed transmission”; define HGT criteria; use longitudinal/strain tracking or transfer assays when claiming HGT	Inflates Level 2 → Level 3/4
Unversioned/outdated antibiotic-resistance genes databases and unclear alignment thresholds	Results are not reproducible and can change with database updates	Report database name + version/date; tool versions; identity/coverage thresholds; consider cross-tool concordance	Undermines Level 1 validity

To make these contributions falsifiable and checkable, we advance three concrete claims. First, mechanistic language in metagenomic AMR studies should be explicitly tied to an evidence level (i.e., the presence, context, activity, or phenotype of a gene; see What Do We Mean By “Mechanisms” in Metagenomic AMR Studies?) and to allowable wording, rather than inferred from gene presence alone. Second, in most studies, “mechanisms” supported by metagenomics primarily concern ecological dissemination (host/mobile genetic elements context and gene flow), unless the causality of a gene in causing an antibiotic resistance phenotype has been demonstrated. Third, we propose minimum evidence thresholds for host/MGE linkage—i.e., the physical or statistical association between an antibiotic resistance gene and a host taxon or a mobile genetic element, typically inferred from co-localization on the same DNA molecule (e.g., spanning reads, shared contigs, or Hi-C contacts)—and for validation escalation, so that readers can distinguish (i) community-level resistance potential, (ii) context-supported dissemination potential, (iii) activity evidence under stated conditions, and (iv) phenotype/causality. To keep the framework grounded, Figures 1–2 include in-figure callouts (A–C) that tie evidence escalation to representative high-impact studies (19, 20, 23).

## WHAT DO WE MEAN BY “MECHANISMS” IN METAGENOMIC AMR STUDIES?

In the AMR literature, “mechanisms” can refer to (i) molecular resistance determinants (e.g., target modification, enzymatic drug inactivation, efflux), (ii) ecological/evolutionary mechanisms (selection, population structure, and HGT), or (iii) pharmacological modes of action of antimicrobials. Because metagenomics measures DNA, it directly supports (i) and parts of (ii), while (iii) usually requires orthogonal assays.

To reduce over-interpretation, we propose an evidence ladder for mechanistic inference in metagenomic AMR studies (see also Fig. 1):

- Level 1: Presence
- Level 2: Context
- Level 3: Activity
- Level 4: Phenotype/causality

These levels can be used as an interpretation checklist, providing operational minimum criteria as follows.

- Level 1 (presence) describes the detection of antibiotic resistance genes or mobile genetic elements within the metagenomic data set using appropriate controls. This level confirms that a resistance determinant is present in the sample, but it does not identify its host, genetic context, or functional activity. Simply identifying the presence of these genes is not sufficient for host attribution or transmission claims.
- Level 2 (context) indicates that ARGs are linked to their host(s) and to specific genetic neighborhoods. By “linked,” we mean a physical or statistical association established through co-localization on the same DNA molecule—for example, via long reads spanning both the antibiotic resistance genes and a host-taxonomic marker or a mobile genetic element, consistent read-pair support across contigs, Hi-C contact probability, or methylation-based assignment. The genetic context includes whether the genes reside on a plasmid, integron, transposon, operon, or their copy number variations. For Level 2 assignment, studies should report at minimum: (i) the linkage method (assembly/long reads/read-pairs/Hi-C) and its limitations, (ii) explicit confidence rules for linking an antibiotic resistance gene to a host or mobile genetic elements (e.g., long reads spanning both loci, consistent read-pair support, or a defined Hi-C association threshold), (iii) contig/MAG quality metrics used to support host assignment, and (iv) how plasmids/integrons/transposons were identified (tool/database versions and decision rules). If these items are missing or weak, conclusions should be restricted to community-level resistance potential without host attribution.
- Level 3 (activity) provides evidence that the resistance determinants are expressed or active under relevant conditions (e.g., targeted transcript or protein readouts). This level demonstrates transcription or protein production under the specific conditions tested, thereby strengthening mechanistic plausibility. However, expression alone does not prove that the determinant confers a resistance phenotype in the original host or in clinical settings. The condition and assay context must be stated (i.e., what was measured, in which samples/conditions, and with what controls), and activity claims should be limited to “activity under the conditions tested.”
- Level 4 (phenotype/causality) requires that cultured isolates, functional metagenomics, or genetic experiments (e.g., cloning, knockout, or transfer assays) demonstrate that the determinant confers resistance and explains the phenotype. This is the highest level, closing the genotype-to-phenotype loop. At least one phenotype-anchored validation route must be described, with clear mapping between the genetic determinant and the observed resistance phenotype.

## CORE APPLICATIONS OF METAGENOMICS IN AMR RESEARCH

### Resistome surveillance across environments

Shotgun metagenomics provides a quantitative view of antibiotic-resistance gene abundance and diversity across reservoirs such as wastewater, agriculture-impacted soils, built environments, and clinical cohorts (11–15). Wastewater treatment plants are repeatedly identified as hotspots where high microbial density and frequent exposure to antibiotics and disinfectants can co-select for resistance and promote exchange via mobile genetic elements (11, 12). Metagenomics is particularly informative when paired with metadata (antibiotic usage, effluent treatment steps, and seasonality) and when normalization strategies are explicitly reported (per cell, per 16S, per biomass, or per sample volume).

In synthesis articles that aim for the conceptual clarity and methodological depth typical of journals such as Nature Reviews Microbiology, surveillance findings are most interpretable when the measurement model is explicit: (i) whether antibiotic-resistance genes abundance is estimated by read-based mapping, assembly-based annotation, or hybrid strategies, (ii) how multi-copy genes and fragmented hits are handled, and (iii) whether abundance is interpreted as relative composition (within-sample proportions) or as an attempt at absolute load (per biomass/volume/cell equivalents). These choices can change qualitative conclusions even when the same samples are analyzed, and they should be treated as part of the study design rather than an implementation detail (5, 6, 16, 17).

A visual pipeline for these interpretation checkpoints is shown in Fig. 2.

Interpretation checkpoint: in surveillance papers, the strongest defensible claim is often about “resistome burden and composition under the stated quantification scheme,” not about selection or exposure-driven causality. Where causal language is desired, studies should pre-specify the comparison framework (time-series, intervention, or exposure gradient), document batch structure and negative controls, and report sensitivity checks (e.g., alternative database/parameter sets) that show conclusions are robust (16–18).

Interpretation checkpoint: Are reported resistome differences robust to compositional effects, biomass variation, and batch structure? Studies should state a normalization strategy and uncertainty/sensitivity checks; otherwise, restrict claims to descriptive patterns rather than selection or exposure-driven mechanisms. Where feasible, internal standards (spike-ins) or cell-count-linked normalization can help separate true abundance shifts from sampling and extraction variability.

### Linking antibiotic-resistance genes to hosts and mobile genetic elements: from catalogs to gene flow

A major limitation of short-read metagenomics is fragmentation: antibiotic-resistance genes are detected, but their hosts and mobile genetic elements remain ambiguous. Long-read sequencing and improved genome-resolved metagenomics have strengthened the ability to reconstruct metagenome-assembled genomes (MAGs) and to resolve plasmid- and integron-associated antibiotic-resistance gene neighborhoods (8, 19–21). This “context-first” view is essential for mechanistic claims about dissemination (e.g., whether an antibiotic-resistance gene is embedded in a class 1 integron, carried on a broad-host-range plasmid, or chromosomally fixed in a lineage).

Even with long reads, HGT is typically inferred rather than directly observed. Robust inference benefits from longitudinal sampling, strain-resolved tracking, and explicit criteria for calling transfer versus clonal spread (9). Claims should distinguish between “potential mobility” (mobile genetic element signatures present) and “observed transmission” (supported by time-series, epidemiological linkage, or experimental transfer assays).

Methodological synthesis (what reviewers look for): host–antibiotic-resistance gene linkage is not a single technique but a bundle of assumptions. Short-read studies most often rely on contig co-localization (antibiotic-resistance genes and host marker genes on the same contig/bin), read-pair support across contigs, and binning-based assignment to MAGs; each is vulnerable to chimeric assembly, strain heterogeneity, and misbinning. Long reads can substantially strengthen linkage when individual reads span an antibiotic-resistance gene and a host-anchoring locus or when they resolve complete plasmids; however, long-read error profiles, DNA extraction biases, and plasmid multiplicity can still complicate interpretation (8, 19–21). Complementary linkage signals (e.g., methylation-based association in native long reads) can provide additional support for plasmid–host pairing when spanning evidence is limited (22).

Operational recommendation: papers that make dissemination claims should report a “linkage evidence table” (even in supplements) specifying (i) the linkage signal type(s) used, (ii) pre-specified thresholds (e.g., minimum spanning read length, minimum number of supporting read pairs, and Hi-C association thresholds if used), (iii) MAG/contig quality metrics used for host assignment, and (iv) failure rates under alternative assemblies/filters. Table 1 provides a compact template for these items that reviewers can quickly audit. This converts otherwise narrative linkage into checkable evidence consistent with Level 2.

To make Level 2 claims more reproducible across studies, we recommend that the linkage evidence table includes a minimal set of quantitative fields rather than prose only: the number of spanning reads supporting each key antibiotic-resistance gene–host/mobile genetic element linkage (and the minimum span length across the junction), the number of supporting read pairs (and whether insert size/orientation are consistent with the linkage), the linkage stability under reasonable alternative assemblies/binning filters (e.g., the fraction of top linkages retained), and—if proximity ligation is used—the association statistic and threshold definition (plus how negatives/decoys were handled). Reporting these fields does not guarantee correctness, but it makes linkage strength auditable and helps prevent “context” claims that are irreproducible.

Interpretation checkpoint: What is the concrete evidence for host/mobile genetic element linkage (reads spanning loci, consistent read-pairs, and Hi-C thresholds), and how often does the linkage survive alternative assemblies/filters? If linkage criteria are not explicit, host attribution and dissemination narratives should be downgraded to community-level resistance potential (23).

### Novel determinants and functional metagenomics

Metagenomic annotation against reference databases can miss divergent antibiotic-resistance genes. Functional metagenomics addresses this by cloning environmental DNA into expression hosts and screening for resistance phenotypes, enabling discovery of previously unknown determinants and broadening reference sets used for annotation (10, 15). When integrated with sequencing, functional screens provide Level 4 evidence that closes the loop from genotype to phenotype.

Interpretation checkpoint: Does the functional screen demonstrate resistance in a defined host/condition, and is the sequence-to-phenotype mapping unambiguous? Key limitations include host-expression bias, cloning context effects, and selection conditions; manuscripts should avoid overgeneralizing phenotype across taxa/ecosystems without follow-up validation.

In comprehensive reviews, it is useful to separate two discovery outcomes that are often conflated: (i) discovery of new sequence space (novel families or divergent variants) that improves Level 1 detection sensitivity after database backfilling, versus (ii) discovery of transferable resistance potential in clinically relevant hosts or mobile genetic elements (which still requires Level 2 context linkage and/or Level 4 validation in appropriate hosts). In other words, functional metagenomics can deliver Level 4 evidence for the screened host/conditions, but ecological and clinical generalization requires additional steps and should be stated explicitly.

### Clinical metagenomics: rapid identification and resistance determinants

Metagenomic next-generation sequencing (mNGS) supports broad pathogen detection in difficult-to-diagnose infections and can identify resistance determinants in the same assay (24, 25). In practice, clinical actionability depends on sample type, host background, turnaround time, and interpretability of antibiotic-resistance gene signals. Because gene presence does not guarantee phenotypic resistance, clinical reporting is strongest when metagenomic calls are linked to pathogen assignment (host-context) and integrated with local epidemiology and, whenever possible, phenotypic susceptibility testing.

Interpretation checkpoint: Are resistance determinants attributed to the causative pathogen (rather than commensals/background DNA), and is the report aligned to evidence level? Without a confident host-context, clinical wording should be limited to “detected resistance determinants in the sample” and paired with confirmatory AST or targeted assays.

For clinical sections in comprehensive reviews, it is also important to separate two decision layers: (i) diagnosis (pathogen detection and attribution) and (ii) therapy guidance (resistance prediction). Diagnostic accuracy and real-world utility depend on specimen type, host background, timing relative to antibiotics, and the reporting rules used to filter contaminants and low-level signals (21, 24–30). Resistance prediction is typically most defensible when resistance determinants are (i) confidently linked to the pathogen and (ii) interpreted in a local epidemiological context that calibrates genotype–phenotype expectations.

### Representative case studies: what metagenomics uniquely enables

To make the evidence ladder concrete, three recent high-impact examples illustrate where metagenomics is not merely a cataloging tool, but enables inferences that are difficult to obtain with culture-only workflows.

Case A (8): using a combination of Nanopore and PacBio long-read sequencing, Qian et al. profiled the antibiotic resistome in feces from a ceftiofur-treated cow following antibiotic enrichment. By assembling complete or near-complete plasmid sequences and draft bacterial chromosomes, the authors directly captured physical co-localization of antibiotic-resistance genes with mobile genetic elements (e.g., insertion sequences, transposons, and integron-associated integrases) on the same DNA molecules. This approach enabled host assignment of antibiotic-resistance genes-carrying contigs through taxonomic classification of assembled sequences, revealing that Enterobacteriaceae were the dominant hosts of plasmid-borne resistance genes, including clinically important ESBL genes (CTX-M, TEM, CMY, and CARB) physically linked to aminoglycoside, sulfonamide, and mercury resistance genes. This study provides Level 2 (context) evidence: it demonstrates which antibiotic-resistance genes co-occur with which mobile genetic elements and which bacterial taxa carry them, but does not directly measure horizontal transfer events. The authors appropriately conclude “potential mobility” rather than confirmed transmission.

Case B (9): in a 2-year prospective study across four hospital wards, León-Sampedro et al. combined epidemiological data from >9,000 patients with whole-genome sequencing of 250 pOXA-48-carrying enterobacterial isolates. Using statistical transmission models and the structured coalescent-based tool SCOTTI, they distinguished between two dissemination routes: between-patient transmission of resistant clones and within-patient horizontal transfer of the carbapenemase-encoding plasmid pOXA-48. High-risk *Klebsiella pneumoniae* ST11 was identified as the main driver of between-patient spread, with specific rooms acting as transmission hotspots. Crucially, by tracking rare plasmid single-nucleotide polymorphisms as genetic fingerprints across isolates from the same patient, the authors provided strong evidence for within-patient plasmid transfer from *K. pneumoniae* to *Escherichia coli*, *Citrobacter freundii*, and other enterobacteria. This study reaches Levels 2 and 3: it moves beyond static context to infer *in vivo* plasmid dissemination dynamics using temporal and strain-resolved data. However, definitive directionality and causality would require direct experimental transfer assays or stronger epidemiological linkage.

Case C (10): Gschwind et al. constructed ResFinderFG v2.0, a curated database of antibiotic resistance genes discovered through functional metagenomics. They systematically screened 50 publications, retrieved 23,776 insert sequences from functional selections, applied stringent curation steps (consistency between predicted antibiotic-resistance genes and selecting antibiotic, protein size filters, removal of inserts with multiple antibiotic-resistance genes), and obtained 3,913 unique antibiotic-resistance genes. Unlike conventional databases that rely on sequence homology to known antibiotic-resistance genes from culturable pathogens, ResFinderFG v2.0 captures antibiotic-resistance genes from non-culturable and non-pathogenic bacteria that share low identity with database entries. Comparative analysis against the Global Microbial Gene Catalog showed that ResFinderFG v2.0 enabled detection of antibiotic-resistance genes—particularly those conferring resistance to beta-lactams, glycopeptides/cycloserine, and sulfonamides/trimethoprim—that were missed by other databases. This work provides Level 4 (phenotype/causality) evidence for the screened host/conditions, as each entry is functionally validated to confer resistance in a defined experimental system. However, ecological and clinical generalization (e.g., activity in non-lab strains or natural microbiomes) would require additional validation.

## PRACTICAL LIMITATIONS THAT DRIVE CONFUSION—AND HOW TO ADDRESS THEM

### Culture-dependent methods are not obsolete

Culture-independent sequencing expands access to uncultured diversity, but cultivation provides phenotypes, strain collections, and causal tests. For AMR, hybrid strategies—metagenomics to map candidates and cultivation/functional assays to validate—consistently yield the most defensible mechanistic insights.

### Contamination, batch effects, and host DNA

Low-biomass samples are especially vulnerable to reagent contamination (“kitome”), which can distort community composition and resistome profiles (26). Studies should report negative controls, extraction blanks, and thresholding rules. In clinical tissues, host DNA often dominates reads; host depletion and careful interpretation are necessary to avoid false negatives and misassigned antibiotic-resistance genes.

To make this section operational, we recommend that manuscripts explicitly list (i) negative controls processed end-to-end (extraction blanks and library blanks), (ii) positive controls (mock communities or reference materials) that verify pipeline sensitivity, and (iii) batch-handling strategies (randomization, blocking, and whether batches are confounded with clinical groups). Where host background is high, host depletion or targeted approaches can improve sensitivity, but they introduce their own biases (differential lysis, loss of microbial DNA, and altered representation of plasmids versus chromosomes). The practical consequence is that “no antibiotic-resistance genes detected” is rarely interpretable without reporting the read budget, host fraction, and detection limits under the exact workflow (17, 26, 31, 32).

Interpretation checkpoint: if contamination-aware filtering or host removal steps are opaque (software, thresholds, and decision rules not provided), conclusions about low-abundance pathogens/antibiotic-resistance genes should be downgraded. In a review context, this is a recurring reason why clinical mNGS studies are difficult to compare across sites.

### Database and pipeline dependence

Taxonomic and antibiotic-resistance gene calls depend strongly on database choice, parameter settings, and classifier/aligner behavior, with substantial variability across tools (16, 17). For reproducibility, studies should report versions of databases, software, and key thresholds; whenever feasible, share workflows and include sensitivity analyses.

In practical pipelines, this typically includes taxonomic profiling (e.g., Kraken 2 or marker-gene-based profilers such as MetaPhlAn2) and sequence alignment/mapping engines used for annotation and context reconstruction (e.g., DIAMOND for protein-level searches and Minimap2 for long-read alignment) (33–36).

From a reviewer’s standpoint, the central issue is that “the pipeline is part of the result.” Tool families make different tradeoffs (speed, sensitivity to novel sequence space, robustness to contamination, and interpretability of confidence scores), and database curation choices can shift labels and abundance estimates. Therefore, comprehensive papers increasingly treat pipeline evaluation as a first-class analysis: they (i) justify database selection and update cadence, (ii) report identity/coverage cutoffs and how multi-mapping reads are handled, and (iii) demonstrate robustness to reasonable alternative settings (16–18). When space is limited, a minimal sensitivity analysis can be as simple as re-running key comparisons under an alternative database/threshold pair and reporting whether directional conclusions change.

### From “antibiotic-resistance genes detected” to resistance mechanisms

Overstatement is common when metagenomic findings are described as “mechanisms.” Using the evidence ladder (see What Do We Mean By “Mechanisms” in Metagenomic AMR Studies?) helps ensure that mechanistic statements are supported by context, activity, and phenotype.

### Minimum reporting checklist for defensible mechanistic inference

One recurring challenge in metagenomic AMR studies is that key methodological details (controls, thresholds, database versions, and host-linking criteria) are omitted, making it difficult to judge whether Levels 1 and 2 evidence is reliable and whether claims are proportional to evidence. Table 1 lists a practical “minimum reporting checklist” aligned to the evidence ladder. Figure 2 outlines an end-to-end workflow with decision gates that link data types to allowable claims.

Practical definitions of the decision gates (Fig. 2):

- Gate A (sample validity): Are negative controls, batch structure, read budget, host fraction, and detection limits sufficiently reported to interpret absence/low-abundance signals?
- Gate B (context/linkage): Is there explicit, thresholded evidence linking antibiotic-resistance genes to host(s)/mobile genetic elements (and are linkage robustness checks reported)? If not, restrict conclusions to Level 1 presence.
- Gate C (activity under conditions tested): Is activity assessed with a stated assay, condition context, and controls (and is wording limited to those conditions)?
- Gate D (phenotype anchoring): Is there genotype-to-phenotype validation (culture AST linked to the determinant, functional metagenomics, genetics, or transfer assays) sufficient for Level 4 language?

To make this section operational for critical appraisal, Table 2 summarizes frequent pitfalls as a “Pitfall → consequence → minimum fix” checklist, mapped to the evidence ladder level at which the pitfall most commonly inflates claims.

## OUTLOOK

Metagenomics has matured from a community profiling method into a framework for AMR surveillance and hypothesis generation. The next leap in scientific value will come less from expanding catalogs and more from raising the evidentiary standard for mechanistic inference—especially by linking antibiotic-resistance genes to hosts and mobile genetic elements, integrating time-series designs, and prioritizing functional validation. When metagenomics is positioned as complementary to cultivation and experiments, it provides a rigorous path from community-scale observation to actionable AMR mechanisms.

Looking forward, three practical shifts can increase both scientific value and interpretability. First, studies should pre-specify the intended claim level (Levels 1–4) and use decision gates (Fig. 2) to keep conclusions aligned with available evidence. Second, reporting should move toward minimal, checkable standards (Table 1) and explicit handling of common pitfalls (Table 2), enabling cross-study comparison and meta-analysis. Third, investment in validation and calibration—reference materials, negative-control-aware workflows, and hybrid designs that connect metagenomes to isolates/phenotypes—will determine whether metagenomics contributes primarily to surveillance or to mechanism-level understanding.

## ENABLING TECHNOLOGIES AND ANALYTICAL FRONTIERS (TOWARD A MORE COMPREHENSIVE REVIEW)

The evidence ladder (Fig. 1) clarifies what kinds of claims are supported by different evidence types, but it does not prescribe a single technology stack. In practice, the field is moving quickly, and several innovations now influence what can be measured, linked, and validated. Below, we highlight developments that expand capability while explicitly mapping them to the evidence ladder to reduce over-interpretation.

To keep this forward-looking section actionable, Table 3 summarizes selected resources and method classes that recur in AMR metagenomics, and Table 1 offers a “minimum, checkable” analysis-and-reporting bundle that helps align computational outputs to claim language and evidence levels.

**TABLE 3:** Selected bioinformatics resources and AI-enabled methods that frequently appear in AMR metagenomics studies

Resource/method	Category	What it is used for in AMR metagenomics	Key reference(s)
Deep antibiotic-resistance genes	Database + model	Learning-based antibiotic-resistance genes prediction from short reads and full-length sequences; improves sensitivity beyond strict best-hit rules	37
Reference materials/benchmarking studies	Standardization	Quantifies variability across pipelines and laboratories; supports more reproducible evidence-level assignment	17, 26, 31, 32
DNA methylation linkage (native long reads)	Host–mobile genetic elements linkage	Associates plasmids/mobile genetic elements to host genomes using methylation motifs; strengthens Level 2 context when spanning evidence is present	22
Single-microbe/single-cell genomics	Strain resolution	Links antibiotic-resistance genes and genomic context to individual cells/strains rather than community averages	38, 39
CRISPR enrichment/depletion	Targeted wet-lab method	Enriches rare regions (e.g., integrons) or depletes host background to improve sensitivity	40, 41
Metagenome mining + ML for AMPs	Discovery	Expands candidate antimicrobial repertoires from global metagenomes; requires experimental validation for Level 4 claims	14, 42
AlphaFold 3	Structure prediction	Supports rapid structural hypothesis generation and interaction modeling to prioritize candidates for validation	43

### Antibiotic-resistance genes annotation beyond “best-hit”: databases, thresholds, and learning-based models

Antibiotic-resistance genes profiling is sensitive to database choice, update cadence, and alignment/classification thresholds (16, 17, 44, 45). In practice, database selection is often operationalized via widely used curated resources (e.g., CARD, ResFinder, and AMRFinder), each with its own curation scope, reference sets, and update cycles (46–48). For comprehensive reporting, studies increasingly pair traditional alignment-based annotation with sensitivity analyses (multiple databases and multiple thresholds) and/or learning-based predictors. Deep-learning approaches, such as Deep antibiotic-resistance genes, have been used to improve antibiotic-resistance genes prediction from short reads and full-length sequences, with curated outputs feeding back into database expansion (37). Complementary efforts aim to provide reference data sets for environmental antibiotic-resistance genes abundance and to support more standardized comparisons across studies (49).

In a comprehensive review context, it is also helpful to distinguish “metagenomics as measurement” from “metagenomics as discovery.” Functional metagenomics provides a phenotype-anchored route to discover novel determinants that are absent from sequence databases and can backfill reference sets used by purely *in silico* annotation (15, 50, 51).

Interpretation checkpoint: learning-based antibiotic-resistance genes prediction can strengthen Level 1 detection sensitivity, but it does not automatically upgrade claims to Levels 2–4. Host/mobile genetic element context and phenotype validation are still required for mechanistic language.

### Benchmarking, reference materials, and inter-laboratory reproducibility

Metagenomic results can vary substantially across laboratories, platforms, and pipelines, particularly for low biomass or host-dominated clinical samples (17, 26, 31, 32, 52). Multicenter evaluations and assay validation studies highlight that analytical variability can be large enough to mask biological signals if controls, thresholds, and reporting standards are not explicit (31, 33). In parallel, clinical meta-analyses and prospective studies emphasize that diagnostic accuracy depends on sample type, workflow, and how results are interpreted and reported to clinicians (27–29).

Beyond technical variability, a comprehensive AMR perspective should also keep ecological selection pressures in view. Sub-inhibitory antibiotic and biocide exposures can promote horizontal transfer and persistence of resistance determinants; metagenomics can track these signatures at scale, but mechanistic claims still require explicit criteria and (where feasible) validation (32, 53–56).

Cost and operational constraints can be equally determinative of what becomes “standard.” Practical advances in library preparation and workflow optimization can enable broader surveillance and benchmarking (38, 57–59). In clinical contexts, large, carefully phenotyped cohorts and deep diagnostic workups (including central nervous system infections) provide essential ground truth for interpreting what metagenomic detection does—and does not—support (27–30).

Interpretation checkpoint: standardization and validation improve confidence within a given evidence level; they do not replace Level 4 phenotype anchoring when the claim is “confers resistance.”

### Long reads, epigenetic linkage, and single-cell resolution for host–mobile genetic elements attribution

Long-read sequencing continues to improve genome-resolved assembly and antibiotic-resistance genes-context reconstruction (8, 19–21). A key advance for dissemination mechanisms is the ability to associate plasmids and other mobile genetic elements with their hosts more directly. DNA methylation patterns preserved in native long-read data have been used as “barcodes” to link plasmids to host genomes in complex communities, strengthening Level 2 host/mobile genetic elements attribution when spanning evidence is present (22). In parallel, single-cell and single-microbe genomics can resolve strain-level structure and link genes to individual cells rather than community averages, which can sharpen mechanistic narratives about reservoirs and dissemination when designs are appropriately controlled (39, 60).

As communities and reference sets expand, consistent taxonomic frameworks also matter for comparability across studies. Domain-to-species taxonomies derived from genome-resolved data (e.g., GTDB) help reduce naming inconsistencies that otherwise complicate host attribution and meta-analysis across cohorts (40).

### CRISPR-enabled enrichment/depletion for targeted questions

CRISPR-based strategies are increasingly used to enrich rare genomic regions (e.g., integrons) or deplete abundant host/rRNA background to improve sensitivity in both environmental and clinical contexts (41, 42). These approaches can improve feasibility and turnaround time, but the interpretation still depends on controls and on how linkage and phenotype evidence are established.

### AI-enabled discovery and “closed-loop” workflows

AI is influencing AMR metagenomics primarily by (i) improving detection/annotation (see Antibiotic-resistance Genes Annotation Beyond “Best-Hit”: Databases, Thresholds, and Learning-based Models) and (ii) accelerating hypothesis generation from large-scale metagenomic sequence collections. In this review’s evidence ladder, these contributions mostly strengthen Level 1 (what might be present) and increase throughput for candidate prioritization; they do not, by themselves, provide host/mobile genetic elements context (Level 2) or phenotype anchoring (Level 4).

In a comprehensive AMR review, AI-enabled mining/design is included here as a complementary “countermeasure” track: it can help discover or prioritize candidate antimicrobials and intervention hypotheses from metagenomic sequence space, but it should not be conflated with evidence for resistance mechanisms or clinical resistance prediction.

**Fig 3:**
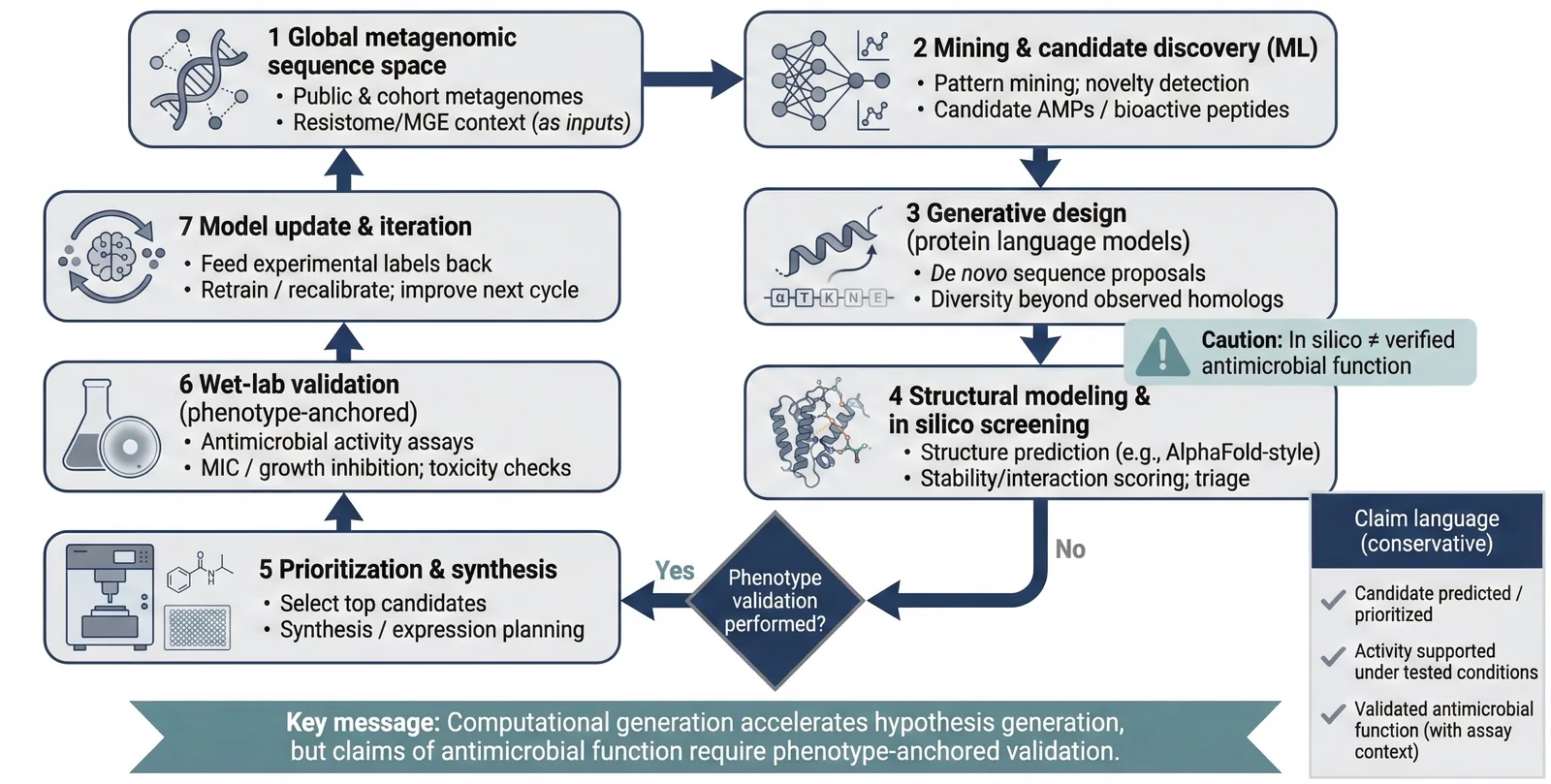
The AI-driven closed-loop for antimicrobial discovery from metagenomes. Conceptual workflow illustrating how large-scale metagenomic mining can feed candidate discovery (e.g., antimicrobial peptides), how generative models propose new sequences beyond observed diversity, how structure prediction and *in silico* screening prioritize candidates, and how wet-lab validation returns data to improve subsequent design cycles. The key point is that computational generation accelerates hypothesis generation but still requires phenotype-anchored validation to claim antimicrobial function and resistance-relevant mechanisms (43, 61).

As an illustration of AI-enabled mining with selective experimental follow-up, AMPSphere used machine learning to mine global metagenomes and reported a large catalog of candidate antimicrobial peptides with experimental validation of selected hits (43). Structure prediction tools such as AlphaFold 3 can further support prioritization by enabling high-accuracy modeling of biomolecular interactions from sequence, but in this context, they should be interpreted as hypothesis-generating unless coupled to condition-matched functional assays (61). Figure 3 sketches a “closed-loop” pattern (mining/design → prioritization → wet-lab testing → model improvement); within AMR metagenomics, the loop upgrades the evidentiary level only when validation steps explicitly connect sequences to function and phenotype.

Interpretation checkpoint: treat AI-enabled mining/design as candidate generation; do not use it to justify mechanistic language without explicit linkage (Level 2) and phenotype-anchored validation (Level 4).

## Data Availability

This study is a review and did not generate new primary data. Data discussed in this manuscript are available from the cited literature and public databases.
